# Lactic acid produced by optimal vaginal *Lactobacillus* spp. potently and specifically inactivates HIV-1 *in vitro* by targeting the viral RNA genome and reverse transcriptase

**DOI:** 10.1371/journal.ppat.1013594

**Published:** 2025-10-10

**Authors:** Muriel Aldunate, David Tyssen, Adam Johnson, Catherine F. Latham, Paula Ellenberg, Nathan Cowieson, Joshua A. Hayward, Rob J. Center, Paul A. Ramsland, Anna C. Hearps, Gilda Tachedjian

**Affiliations:** 1 Life Sciences Discipline and Disease Elimination Program, Burnet Institute, Melbourne, Victoria, Australia; 2 Department of Microbiology, Monash University, Clayton, Victoria, Australia; 3 Australian Synchrotron, Clayton, Victoria, Australia; 4 Department of Microbiology and Immunology at the Peter Doherty Institute for Infection and Immunity, University of Melbourne, Melbourne, Victoria, Australia; 5 School of Science, RMIT University, Melbourne, Victoria, Australia; 6 Department of Immunology, Monash University, Melbourne, Victoria, Australia; 7 Department of Surgery, Austin Health, The University of Melbourne, Heidelberg, Victoria, Australia,; 8 Department of Infectious Diseases, Monash University, Melbourne, Victoria, Australia; 9 Department of Infectious Diseases, University of Melbourne, Melbourne, Victoria, Australia; NIH, NIAID, UNITED STATES OF AMERICA

## Abstract

Vaginal microbiota modulates susceptibility to sexually transmitted infections and produces carboxylic acid metabolites that have antimicrobial activity; however, their activity against viral sexually transmitted infections is not well defined. We determined the HIV-1 virucidal activity of lactic acid (LA), short chain fatty acids (SCFAs), and succinic acid, representing conditions observed in women with an optimal *Lactobacillus*-dominated vaginal microbiota compared to women with bacterial vaginosis. Virucidal activity against enveloped HIV-1 and HSV-2, the non-enveloped HPV16, and the mechanism by which LA inactivates HIV-1 was further assessed. LA was > 10-fold more potent at inactivating an HIV-1 transmitted/founder strain than SCFAs and succinic acid when tested at an equivalent 20  mM of protonated acid (p≤0.05). While LA decreased HIV-1 infectivity by >10^3^-fold, virions were intact, expressed a similar gp120:p24 ratio, and showed only a 2-fold decrease in CD4 binding compared to untreated HIV-1 (p≤0.05). Treatment of recombinant gp120 with LA revealed no major conformational changes by small angle X-ray scattering. LA treatment of HIV-1 resulted in an 80% decrease in virion-associated reverse transcriptase activity compared to untreated virus (p < 0.01), which was more potent than acetic acid or HCl-adjusted media at the same pH, with this effect observed in the presence of cervicovaginal fluid. LA decreased HIV-1 virion-associated RNA levels by ∼50% compared to untreated virus (p < 0.001), acetic acid or HCl acidified media. In contrast, HSV-2 virucidal activity of LA was similar to acetic acid and HCl-acidified media while HPV16 was acid-resistant. Our results demonstrate LA’s potent and specific HIV-1 virucidal activity compared to SCFAs and succinic acid found in the female reproductive tract, and its HIV-1 virucidal mechanism mediated by penetration of the viral membrane and core to target a key viral enzyme and nucleic acid. These findings have implications for the vaginal transmission of HIV to partners and neonates during birth.

## Authors summary

When a women’s vagina is colonised with beneficial bacteria called lactobacilli, she has a lower risk of being infected with sexual transmitted infections (STI) including HIV as well as transmitting HIV to her male partners or newborns compared to women with bacterial vaginosis (BV), a common vaginal dysbiosis that increases STI risk. Vaginal bacteria produce products that have biological effects on the vaginal environment; however, the ability of these products to inactivate viral STIs including HIV is unknown. Here we show that lactic acid, a major metabolite produced by beneficial lactobacilli, potently and specifically inactivates HIV-1 compared to other acids that are elevated in vaginal fluid of women with BV. The potent HIV-1 inactivation by lactic acid was not due to viral particle disruption but was mediated by targeting a viral enzyme and the RNA genome critical for HIV-1 infectivity, requiring lactic acid to penetrate the viral lipid envelope and virion core. In contrast, lactic acid lacked specific killing activity against other viral STIs. Our findings provide important mechanistic insights into the virucidal activity of vaginal microbiota metabolites against viral STIs and indicate potential strategies for microbiome-based interventions to prevent HIV.

## Introduction

Heterosexual transmission accounts for the majority of HIV infections worldwide, yet HIV transmission risk in women following receptive vaginal intercourse is low, estimated at 0.08% [1]. This is due in part to the combined physical and immunobiological defences of the lower female reproductive tract (FRT) against invading pathogens, although pathogenic and commensal microorganisms also influence HIV transmission. Sexually transmitted infections (STIs) including herpes simplex virus (HSV), chlamydia, gonorrhoea, and fungal infections are associated with increased HIV acquisition in women [2–6]. Commensal microbes also influence HIV transmission risk as women with a vaginal microbiota dominated by *Lactobacillus* spp. have an estimated 4.4-7.2-fold lower risk of acquiring HIV as well as a decreased risk of other bacterial and viral STIs as compared to women with vaginal microbiota depleted of lactobacilli [7–10]. In women with HIV-1, a vaginal microbiota dominated by lactobacilli is associated with decreased levels of vaginal HIV-1 RNA and a reduced risk of HIV-1 transmission to male sexual partners and neonates [7,11–13]. These findings highlight the importance of the vaginal microbiota in modulating HIV acquisition in women; however, the role of the specific microbial components, including their metabolites, in modulating this risk and the mechanisms through which these effects are conferred remain poorly understood.

Unlike the gut, where microbial diversity is advantageous, an optimal lower FRT microbiota in women of reproductive age is predominated by *Lactobacillus* spp., in particular with *L. crispatus*, which is associated with greatest protection against HIV-1 acquisition [7,8,10]. In contrast, bacterial vaginosis (BV), a vaginal dysbiotic condition that represents the most common clinical manifestation of a non-optimal vaginal microbiota, comprises a high load and relative abundance of diverse obligate and facultative anaerobic bacteria such as *Prevotella* spp., *Fannyhessea vaginae* (previously *Atopobium vaginae*) and *Gardnerella* spp. and is depleted of *Lactobacillus* spp. [14]. Globally, BV affects approximately one in three women of reproductive age [15–17] and is associated with increased risk of a range of adverse sexual and reproductive health outcomes including spontaneous pre-term birth [18–21], and acquisition of STIs [7,22] including HIV [2,23,24] compared to women with an optimal *Lactobacillus*-dominated microbiota [2,7,15–24]. The lower FRT microbiota influences HIV transmission through modifying the physical and immunological properties of the cervicovaginal environment. BV is associated with increased genital inflammation, and in some studies, an increase in activated HIV target cells in the cervix [25–31], which likely contributes to a heightened risk of HIV acquisition [25–31]. In contrast, optimal *Lactobacillus* spp. are associated with lower genital tract inflammation and promote epithelial barrier integrity and wound healing [32–34].

A major mechanism by which optimal and non-optimal vaginal microbiota exert these effects on the host and/or other microorganisms is through the production of bacterial carboxylic acid metabolites including lactic acid (LA), an alpha hydroxy acid, the short chain fatty acids (SCFA) acetic acid, butyric acid, and propionic acid, as well as succinic acid [35]. LA, produced by *Lactobacillus* spp., reaches vaginal concentrations of ∼110 mM and acidifies the vagina to a pH as low as 3.5 [36,37]. Vaginal acidification through LA production is regarded as a key defence in the lower FRT, generating an environment inhospitable to most exogenous microorganisms [38]. In the context of an optimal vaginal microbiota where the pH is ≤4.5, LA is predominantly found in the biologically active protonated (uncharged) form [39]. In contrast, women with BV have a vaginal pH > 4.5, where the biologically inactive lactate anion (charged) form dominates [40].

We and others have shown that protonated LA elicits a range of protective responses from cervicovaginal epithelial cells including maintaining an anti-inflammatory state and enhancing epithelial barrier integrity [34,41–43]. In addition, protonated LA has potent bactericidal and virucidal properties. LA inactivates 17 different BV-associated bacteria [44], *Chlamydia trachomatis* [45], *Neisseria gonorrhoeae* [46], but not vaginal *Lactobacillus* spp. [44], and is virucidal against herpes simplex virus (HSV) [47,48] and HIV (HIV-1 and HIV-2), which is irreversible, as demonstrated *in vitro* [40,49] and *ex vivo* in cervicovaginal fluid (CVF) [39]. LA exists as L- and D-isomers [50,51], with L-LA exhibiting more potent HIV-1 virucidal activity at threshold concentrations [40]. In contrast, D-LA has greater potency in blocking chlamydia infection of cervicovaginal epithelial cells *in vitro* [52] and is associated with enhanced trapping of HIV-1 particles in cervicovaginal mucous from women colonised with a *L. crispatus*-dominated vaginal microbiota [53,54]. Levels of endogenous LA within CVF from women with an optimal vaginal microbiota correlate positively with HIV-1 virucidal activity [39], suggesting this metabolite contributes to the virucidal properties of CVF from women with an optimal microbiota.

In contrast to the LA-enriched environment of an optimal vaginal microbiota, women with BV exhibit increased vaginal pH > 4.5, reduced vaginal concentrations of LA, including protonated LA, and a concomitant increase in SFCAs (i.e., acetic, propionic, butyric acid) and succinic acid [35,55,56]. Certain SCFAs associated with BV elicit heightened production of inflammatory cytokines including TNFα from FRT epithelial cells *in vitro* [42,43], but their effect on the viability of viral STIs such as HIV are not known.

The role of LA and other vaginal microbiota carboxylic acid metabolites and their contribution to BV-associated STI risks, is not fully elucidated. The few studies that have sought to determine the role of SCFAs in BV have primarily focused on their potential immune modulatory [35,42] and barrier abrogating effects [43] on FRT epithelial cells. The study of their potential bactericidal or virucidal properties has been neglected even though many STI causing viruses, including HIV, are known to be acid labile [57–60]. In addition, the mechanism of viral inactivation and the discrete virucidal effects of specific carboxylic acids found in vaginal fluid as compared to low pH alone (HCl adjusted) have not been investigated. Here, we compared the virucidal activity of LA and BV-associated SCFA and succinic acid simulating physiological conditions and investigated the mechanisms through which LA inactivates HIV-1. The findings of this study are anticipated to inform the design of therapeutic modulators of the lower FRT environment to protect women from acquiring and transmitting HIV.

## Results

### LA potently inactivates HIV-1 at concentrations and pH associated with an optimal vaginal microbiota in contrast to SCFA and succinic acid

LA is virucidal against HIV *in vitro* [40], although it is not clear whether this activity is unique to LA or can be mediated and/or potentiated by other carboxylic acid metabolites present in CVF. The L- and D- stereoisomers of LA are produced preferentially by different *Lactobacillus* spp., with *L. crispatus* predominantly producing D-LA while *L. iners* produces only L-LA [50]. We have previously found both isomers possess potent HIV-1 and HIV-2 virucidal activity [40], enhance the cervicovaginal epithelial barrier [34], and mediate immunomodulatory effects [41,42]. Accordingly, here we evaluated a racemic mix of DL-LA.

**Fig 1:**
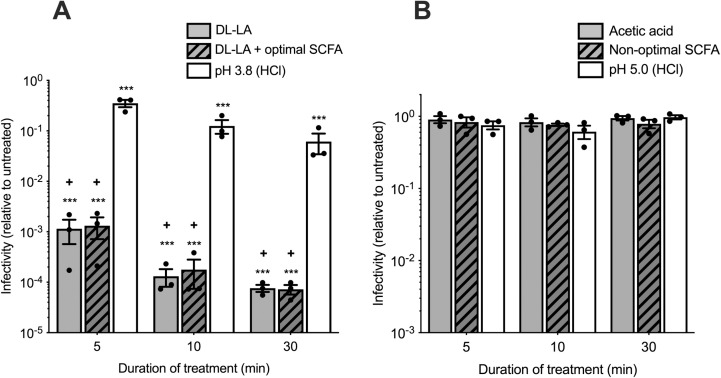
Virucidal activity of LA, SCFA, and succinic acid combinations associated with an optimal vaginal microbiota or BV, representing a non-optimal vaginal microbiota. (A) HIV_RHPA_ was treated with 100 mM DL-LA alone at pH 3.8, DL-LA + optimal SCFA (comprising 100 mM DL-LA, 4 mM acetic acid, 1 mM propionic acid, 1 mM butyric acid, and 1 mM succinic acid adjusted to pH 3.8), representing levels of carboxylic acids present in an optimal, *Lactobacillus*-dominant, vaginal microbiota or HCl-acidified media only [pH 3.8 (HCl)] at 37°C for the indicated times. After incubation, samples were adjusted to neutral pH by 10-fold dilution in DMEM-10 containing 20 mM HEPES, and residual viral infectivity assessed by the TZM-bl infectivity assay. (B) HIVRHPA was treated as in (A) but with 100 mM acetic acid alone at pH 5.0, non-optimal SCFA (comprising 20 mM LA, 100 mM acetic acid, 2 mM propionic acid, 2 mM butyric acid, and 20 mM succinic acid, adjusted to pH 5.0), representing concentrations of carboxylic acids present in women with BV, or HCl-acidified media only [pH 5.0 (HCl)] at 37°C. Figures show viral infectivity relative to the untreated control incubated for the same time. Error bars denote the mean ± S.E.M from n = 3 independent experiments represented by solid black circles. Statistical significance was determined using the unpaired t test where *** denotes p < 0.001 compared to untreated; + denotes p < 0.05 compared to pH 3.8 (HCl) treated samples at the same time point.

Treatment of a subtype B transmitted/founder strain of HIV-1 (HIV_RHPA_) with either DL-LA alone or in combination with a carboxylic acid mixture (DL-LA + optimal SCFA), representative of that found in women with an optimal vaginal microbiota (S1 Table) [35] at a physiologically relevant pH (3.8), elicited rapid and potent inactivation of HIV-1 infectivity (Fig 1A). The DL-LA + optimal SCFA treatment, containing 100 mM DL-LA, decreased virus infectivity by almost 1000-fold relative to untreated virus after only 5 min of incubation and by >10,000-fold after 30 min (p < 0.001 for both; Fig 1A). This profile was similar to HIV-1 inactivation by 100 mM of DL-LA alone (Fig 1A). Notably, both conditions containing DL-LA were dramatically more virucidal than acidity alone at all time points, where media was adjusted to pH 3.8 with HCl [pH 3.8 (HCl) Fig 1A, p < 0.05 for all]. These data indicate that DL-LA, present in the mixture of carboxylic acids simulating concentrations and pH observed in an optimal vaginal microbiota, was largely responsible for the virucidal effect.

We next evaluated the HIV-1 virucidal activity of acetic acid, which is a carboxylic acid present at the highest concentrations in women with BV [35]. Acetic acid was tested either alone, or in combination with a carboxylic acid mixture (Non-optimal SCFA) containing lower levels of LA (20 mM) at pH (5.0) with these conditions representative of that found in women with BV (S1 Table) [35]. No virucidal activity was observed over time following incubation of HIV_RHPA_ with the same molar concentration of acetic acid (100 mM) alone, or with a vaginal carboxylic acid mixture representative of that found in the non-optimal state of BV or with media adjusted to pH 5.0 with HCl alone (Fig 1B). Overall, these data demonstrate that DL-LA, at a physiologically relevant concentration and pH of 3.8, has a marked ability to elicit rapid and potent inactivation of a transmitted/founder strain of HIV-1 irrespective of the presence of other carboxylic acids typically found in CVF of women with an optimal vaginal microbiota. In contrast, under physiological relevant concentrations and pH found in women with BV, acetic acid and/or SCFAs lacked HIV-1 virucidal activity.

### At an equimolar concentration of protonated acid, LA has more potent HIV-1 virucidal activity compared to other carboxylic acids

LA elicited potent HIV-1 virucidal activity when present in a mixture of other carboxylic acids (Fig 1A); however, the protonated concentration of each acid differs based on its unique dissociation constant (pK_a_). To determine if protonated LA has greater HIV-1 virucidal activity than the protonated versions of SCFAs and succinic acid, equimolar concentrations of protonated carboxylic acids at a given pH were calculated using the Henderson-Hasselbalch equation [39]. For these experiments, a threshold, sub-inhibitory acid concentration of 20 mM (protonated form) was chosen to allow differences in virucidal activity of vaginal microbiota carboxylic acids to be assessed. For these and subsequent experiments comparing several different carboxylic acids, a pH of 4.2 was utilised as it is largely compatible with the pKa of the various vaginal acids being assessed while within the pH range present in an optimal vaginal microbiota.

**Fig 2:**
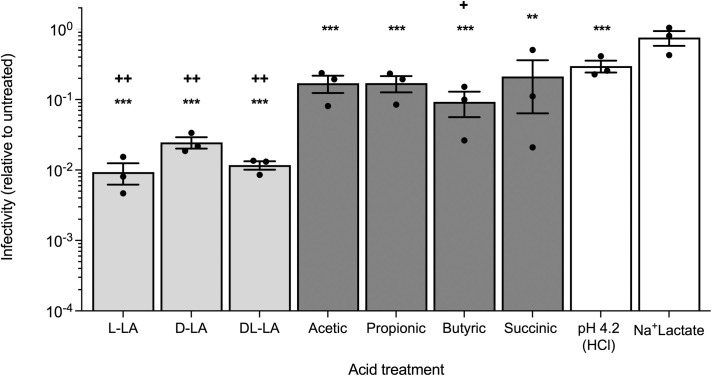
Virucidal activity of equimolar levels of the protonated form of vaginal LA, SCFAs, and succinic acid. HIV_RHPA_ was treated with an equimolar (20 mM) concentration of the protonated form of various carboxylic acids, representing metabolites found in vaginal fluid, at 37°C, pH 4.2 for 30 min. HCl-acidified media only [pH 4.2 (HCl)] and Na+Lactate (pH 7.0) represent low pH and high solute/osmolality controls, respectively. After incubation, samples were adjusted to neutral pH as described in the legend of Fig 1 and viral infectivity determined in the TZM-bl infectivity assay. Viral infectivity is shown relative to the untreated control. Error bars denote the mean ± SEM from n = 3 independent experiments represented by solid black circles. Statistical significance was determined using the paired t test where ** and *** denote p < 0.01 and p < 0.001, respectively compared to untreated; + and ++ denote p < 0.05 and 0.01, respectively compared to pH 4.2 (HCl) treated samples.

Under these conditions, L-, D- and DL-LA substantially reduced viral infectivity by 40–100-fold as compared to untreated HIV_RHPA_ (p < 0.001 for all, Fig 2). Furthermore, the HIV_RHPA_ virucidal activity of all forms of LA (D-LA, L-LA and DL-LA) were significantly greater than the low pH 4.2 control (HCl), which itself caused only a 3-fold reduction in viral infectivity. In contrast, the BV-associated SCFAs acetic, propionic, butyric as well as succinic acid elicited a modest but significant reduction in HIV_RHPA_ infectivity of 4–10-fold compared to untreated virus (p < 0.01 for all; Fig 2) which, except for butyric acid, was not significantly different compared to the low pH 4.2 control, indicating their activity is likely attributable to acidity alone. Sodium lactate (Na+Lactate), employed as a high solute/osmolality and lactate anion control [40], showed no virucidal effects (Fig 2). When adjusted to neutral pH, 100 mM each of L-LA, acetic, propionic, butyric and succinic acid lacked HIV_RHPA_ virucidal activity (S1 Fig), confirming that similar to LA, it is the protonated forms of these acids and not the anion forms that mediates the virucidal effect. These data demonstrate that potency of the virucidal activity of LA is specific, and greater than other vaginal carboxylic acids, low pH alone (HCl), or osmolality.

### LA does not substantially disrupt HIV-1 virion integrity or decrease surface levels of gp120 envelope protein

We next investigated the mechanisms by which LA inactivates HIV-1. A previous study reported that the CCR5-utilising subtype B strain of HIV-1, HIV_BaL_, treated with either 0.3% (w/w) (33 mM) or 1% (w/w) (110 mM) DL-LA and pelleted through a 20% sucrose cushion, followed by Western blot analysis did not show evidence of substantial virion lysis or loss of gp120 from the virion [54]. We have shown previously that HIV_BaL_ is more sensitive to the virucidal activity of L-LA compared to D-LA at concentrations down to 0.3% (w/w) at low pH (pH < 4.5) [39,40]. Accordingly, we investigated the HIV_BaL_ virucidal mechanism of LA using the L-isomer of LA. However, distinct to the previous study that pelleted the virus though a sucrose cushion [54], we examined the effect of L-LA on HIV-1 virion structure and surface envelope proteins by separating treated virus using iodixanol velocity gradient ultracentrifugation. This technique sediments intact viral particles while avoiding contamination with cell-derived microvesicles and viral proteins/debris, which can co-sediment with viral particles during sucrose density-equilibrium gradient or sucrose cushion centrifugation [61]. The effect of L-LA on p24 capsid core protein was assessed to determine whether the virion remained intact after treatment, while the presence of gp120 envelope glycoprotein, which is required for viral entry, relative to p24 was measured to assess gp120 surface protein loss from the viral particle.

**Fig 3:**
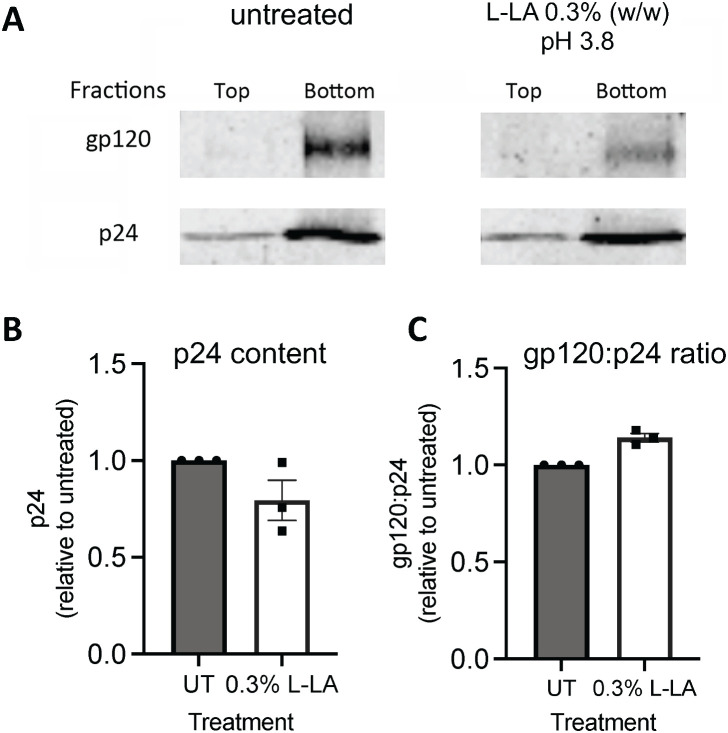
LA has minimal impact on HIV-1 virion integrity and surface levels of gp120 envelope protein. HIV_BaL_ was treated with 0.3% L-LA (33 mM) pH 3.8 for 2 min at 37°C, samples were adjusted to neutral pH as described in the legend of Fig 1, before being subjected to ultracentrifugation through an iodixanol velocity gradient. Five, 2 ml fractions were collected and the top two and bottom three fractions were pooled, treated with Triton-X100, and virion-associated levels of p24 and gp120 assessed by Western blot. (A) p24 and gp120 protein levels in the top 2 and bottom 3 pooled fractions in untreated (left panel) and L-LA treated virus (right panel) from a representative experiment. Combined data for p24 levels (B), and p24:gp120 ratio (C) both determined by quantitative Western blot densitometry of pooled fractions shown in (A). Error bars denote the mean ± S.E.M from n = 3 independent experiments represented by solid black circles and squares. Statistical significance was assessed on data in B and C by paired t-test on raw (non-standardized) values. No significant differences were found for p24 levels (p = 0.54) and the gp120:p24 ratio (p = 0.85) in L-LA treated compared to untreated samples.

Our analyses indicated no significant difference in either the amount of p24 alone (p = 0.54) or the gp120:p24 ratio (p = 0.85) in virions after treatment with L-LA relative to untreated virus (Fig 3), consistent with a previous study [54]. Parallel analysis of viral infectivity following virus treatment with L-LA under the same conditions confirmed a large reduction (>1000-fold) in viral infectivity relative to untreated virus (S2A Fig). These data show that L-LA has minimal effects on surface levels of HIV-1 gp120, indicating that the virucidal activity of L-LA is mediated by other mechanisms.

### LA does not alter the global structure of HIV-1 gp120 and minimally impairs CD4 binding

Given that the HIV-1 gp120 envelope protein remains virion-associated after L-LA treatment and is readily accessible to acid on the surface of the virion, we examined if L-LA’s virucidal activity could be mediated by promoting substantial conformational changes in the envelope protein in solution. Small angle X-ray scattering (SAXS) analysis was therefore employed [62] to assess whether protonated D-LA or L-LA relative to low pH alone (HCl) caused alterations in the global structure of soluble monomeric gp120 protein with regards to molecular size, shape and dimensions.

**Fig 4:**
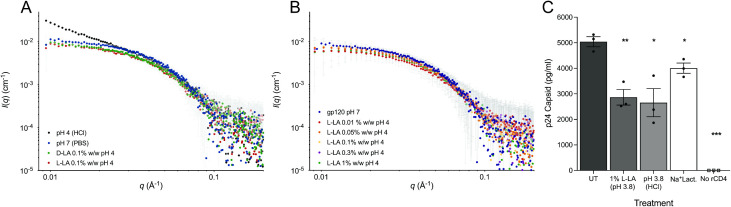
Analysis of L-LA on gp120 protein conformation and rCD4 binding. Global conformational changes in gp120 structure were assessed using SAXS analysis following treatment of recombinant gp120 for 10–15 min at room temperature. (A) Changes in protein structure observed by changes in SAXS scattering curves, where the angle of the scatter, expressed as q (Å-1), was plotted against the intensity of scattered X-rays, I(q) (cm^-1^). Scatter plots of gp120 treated with pH 4.0 (HCl), pH 7.0 (PBS) or 0.1% (w/w) D-LA pH 4.0 or L-LA pH 4.0. (B) as in (A) but with gp120 treated with increasing concentrations of L-LA at pH 4.0 as compared to pH 7, as indicated. The error bars show 2x standard error of the variation in intensity of pixels within bins along the scattering vector Q. (C) Ability of HIV_BaL_ treated with 1% L-LA, HCl (both at pH 3.8) or sodium lactate (Na+Lact) at pH 7.0, to bind recombinant CD4 (rCD4) compared to untreated virus (UT) assessed by ELISA to measure captured virus. Capture rCD4 was omitted in one condition to assess background binding of virus (No rCD4). Error bars denote the mean ± S.E.M from n = 3 independent experiments. Statistical significance was assessed using an unpaired t-test where *, **, and *** indicate p < 0.05, 0.01 and 0.001, respectively as compared to untreated sample.

Our analyses indicate that low pH (HCl) caused denaturation of gp120 protein relative to untreated gp120 at pH 7, as observed by a steep slope in the low q range on the scattering plot [q vs I(q); Fig 4A]. A large signal in this q range indicates that the sample contains a substantial amount of large particles consistent with protein aggregates [63]. In contrast, no aggregation of gp120 was observed following treatment with 0.1% (w/w) (11 mM) D-LA or L-LA at pH 4.0, with the scattering curve being similar to that of untreated gp120 (Fig 4A). To assess the impact of physiologically relevant levels of LA, gp120 was treated with L-LA at a concentration range from 0.01 – 1% (w/w) (1.1 to 110 mM) at pH 4.0 and compared to untreated gp120 at pH 7.0. Our data indicate no detectable changes in the overall structure of monomeric gp120 in solution at any concentration of L-LA tested as determined by SAXS (Fig 4B).

We next investigated whether L-LA elicits conformational changes of HIV-1 gp120 envelope structure, in the context of the virion, that may reduce infectivity by impairing binding of the trimeric gp120 to the cell entry receptor CD4. The ability of virion-associated gp120 to bind recombinant soluble CD4 (rCD4) was assessed using a modified CD4 binding ELISA. Treatment of HIV_BaL_ with 1% (w/w) (110 mM) L-LA (pH 3.8) elicited an almost 2-fold reduction in the ability of HIV-1 to bind to rCD4 relative to untreated HIV-1 (p = 0.004, Fig 4C). However, a similar reduction in rCD4 binding was observed following treatment with low pH alone (HCl, pH 3.8), which was not significantly different to that observed for L-LA (p = 0.75), indicating that the effect was associated with low pH and not specific to L-LA. HIV-1 binding to rCD4 was also slightly but significantly impacted by treatment with sodium lactate, suggesting a potential effect of osmolality and/or the lactate anion (Fig 4C). Analysis of viral infectivity of HIV_BaL_ treated in parallel showed a > 10,000-fold reduction in viral infectivity following treatment with 1% (w/w) L-LA relative to untreated virus (S2B Fig). Overall, these data indicate that L-LA and HCl at pH 3.8 cause a similar, approximately 2-fold reduction in the rCD4 binding ability of HIV-1 gp120. In contrast, L-LA treatment was associated with near complete inactivation of HIV-1 infectivity, suggesting other mechanisms contribute to the potent virucidal activity of LA.

### LA exhibits more potent inhibition of virion-associated HIV-1 RT activity than other acids and retains inhibitory activity in the presence of CVF

Protonated LA present at low pH is membrane permeant [64] and has been reported to inactivate creatine kinase enzyme activity through limited unfolding of protein secondary structure in the absence of aggregation [65]. Thus, we postulated that LA penetrates the HIV-1 lipid envelope and viral core, comprising p24 capsid, to inhibit the function of proteins within the viral core, such as the reverse transcriptase (RT) enzyme [66] that is critical for viral infectivity. To investigate this possibility, HIV_RHPA_ was treated with the same concentration of 37 mM of DL-LA and 37 mM acetic acid at pH 3.8 (representing 20 mM of protonated DL-LA and 33 mM of protonated acetic acid) or 22 mM of acetic acid (representing 20 mM of protonated acetic acid) at pH 3.8. Virion-associated RNA-dependent DNA polymerase activity was assessed using an exogenous template/primer after samples were brought to neutral pH and intact virions subsequently lysed with a non-ionic detergent to release the RT from the virion.

**Fig 5:**
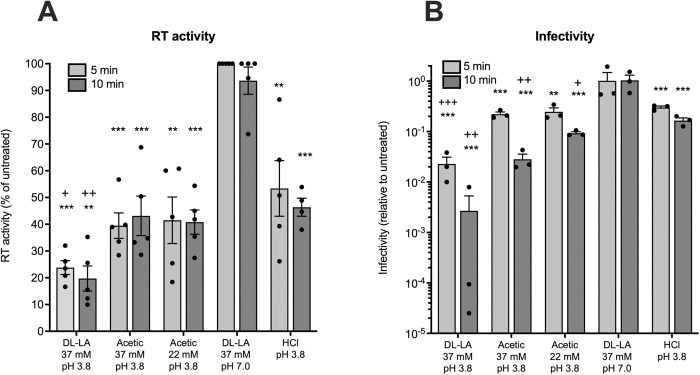
LA is a more potent inhibitor of HIV-1 virion-associated RT activity compared to acetic acid or low pH (HCl) alone. (A) HIV_RHPA_ was treated with 37 mM of DL-LA and 37 mM acetic acid at pH 3.8, representing 20 mM of protonated DL-LA and 33 mM of protonated acetic acid, respectively, or 22 mM of acetic acid (representing 20 mM of protonated acetic acid) at pH 3.8, and HCl at pH 3.8 for 5 min or 10 min at 37^o^C. Virion-associated RT activity of neutralised virus was assessed by enzymatic assay using a radiolabelled substrate, and expressed as a percentage of untreated virus. (B) Viral infectivity was determined in parallel in the TZM-bl infectivity assay and expressed relative to untreated virus. Error bars denote the mean ± S.E.M from at least n = 3 independent experiments represented by solid black circles. Statistical significance was assessed using a paired t-test where ** and *** indicate p < 0.01 and 0.001, respectively compared to untreated; + , ++ and +++ indicate p < 0.05, 0.01, and 0.001, respectively compared to the low pH 3.8 (HCl) control.

Our data show that virion-associated RT activity was markedly decreased upon treatment with DL-LA at pH 3.8, resulting in a 77% and 80% reduction in HIV_RHPA_ RT activity within 5 min (p < 0.001) and 10 min (p < 0.01) of treatment, respectively (Fig 5A). Acetic acid treatment also reduced virion-associated RT activity; however, the reduction in RT activity by DL-LA was significantly more potent than both acetic acid at either the same total concentration (37 mM) or at the same protonated acid concentration (20 mM acetic acid) as DL-LA (p = 0.01 and 0.002, respectively after 10 min of treatment). Treatment with low pH 3.8 alone (HCl) also impaired virion-associated RT activity by approximately 50% (Fig 5A), although the inhibitory activity of DL-LA at the same pH was significantly greater than HCl (p < 0.05 after both 5 and 10 min of treatment). Treatment of HIV_RHPA_ with DL-LA at a neutral pH (7.0) had no detectable effect on virion-associated RT activity (Fig 5A), confirming that the abovementioned effect of LA is specific to the protonated form that predominates at low pH. HIV_RHPA_ infectivity was assessed in parallel and displayed a similar pattern of reduction in viral infectivity (Fig 5B) as observed for virion-associated RT activity (Fig 5A), with a more potent effect of DL-LA as compared to acetic acid and HCl.

To determine if LA treatment inhibits virion-associated HIV-1 RT activity within the context of the cervicovaginal environment in which it is produced, the above analyses were repeated in the presence of CVF pooled from two women with an optimal vaginal microbiota (i.e., without BV) and pooled CVF from two women with BV (non-optimal). HIV_RHPA_ was treated with 20 mM of protonated DL-LA (pH 3.8) in the absence or presence of an equal volume of neat CVF for 5 min at 37^o^C. Following incubation, virions were lysed and subjected to product-enhanced reverse transcriptase (PERT) assay to quantify virion-associated RT activity [67]. In parallel, samples were neutralised to determine viral infectivity. Our data show that LA inhibits virion-associated RT activity in either the absence or presence of CVF with similar inhibitory effects observed with CVF from women with and without BV (S3A Fig). Using PERT, we also showed that inhibition of virion-associated RT activity was less potent when HIV_RHPA_ was treated with media adjusted to pH 3.8 with HCl alone and that no inhibitory effect was observed in the presence of the lactate anion (i.e., Na-Lactate pH 7; S3A Fig). Infectivity data showed a similar pattern to the RT activity data, where treatment with DL-LA in the absence or presence of CVF from women with and without BV both decreased virus infectivity by a ∼1000-fold compared to untreated virus (p < 0.0001; S3B Fig). These data suggest that LA inhibits HIV-1 infectivity by targeting virion-associated RT activity and that this effect is observed in the presence of CVF from women with and without BV.

The inhibitory activity of LA on virion-associated RT could be due to either a direct effect of the membrane permeant acid [64,68] penetrating the virion lipid envelope and core to interact directly with the RT to inhibit its function, and/or due to an indirect effect where LA subtly permeabilises [69,70] the HIV-1 lipid envelope enabling penetration of other factors that inhibit the enzyme. To determine if L-LA treatment can directly inhibit RT enzyme function, we performed experiments with purified recombinant HIV-1 RT. Incubation of recombinant HIV-1 RT with L-LA at low pH demonstrated a dose-dependent impairment of RT activity (S4A Fig). However, a similar level of inhibition was also observed with an equivalent concentration of acetic acid and the low pH (HCl) control (S4B Fig), indicating that recombinant HIV-1 RT protein is highly sensitive to the non-specific inhibitory effect of acidification. Taken together, these data indicate that while low pH alone has a detrimental effect on recombinant HIV-1 RT activity, LA elicits a significantly more potent inactivation of virion-associated RT activity, and this effect is specific to the protonated form of LA that may be better able to penetrate the virion lipid envelope and core compared to acetic acid or protons (H+) to target RT.

### LA promotes degradation of virion-associated HIV-1 RNA

**Fig 6:**
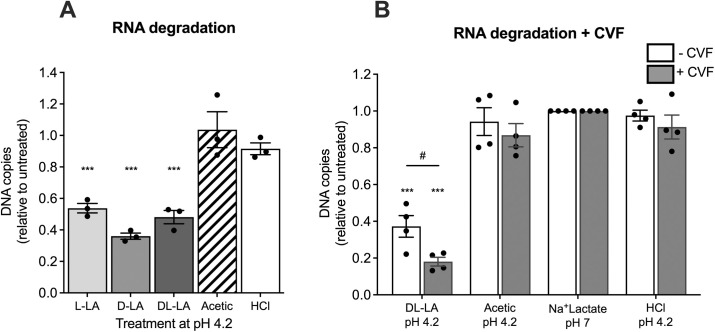
Degradation of virion-associated RNA following acid treatment. (A) HIV_RHPA_ was treated with 20 mM protonated L-lactic acid (L-LA), D-lactic acid (D-LA), racemic lactic acid (DL-LA)(all representing a total concentration of 37 mM of LA), 20 mM of protonated acetic acid or HCl for 5 min (all at pH 4.2) and viral RNA was extracted and amplifiable RNA assessed by qRT-PCR. (B) HIV_RHPA_ was treated with 20 mM protonated DL-LA, 20 mM protonated acetic acid and low pH (HCl) control (all at pH 4.2) as shown in (A), and 37 mM of sodium lactate at pH 7 (Na^+^Lactate pH 7), but in the absence or presence of cervicovaginal fluid (CVF) from women with an optimal vaginal microbiota. Samples were neutralised by dilution in DMEM-10 containing 20 mM HEPES, viral RNA extracted and treated with DNase I, and viral RNA quantified by qRT-PCR using primers directed to the HIV-1 R-U5 region. Error bars denote the mean ± S.E.M of DNA relative to untreated virus from at least n = 3 independent assays, represented by solid black circles. Statistical significance was assessed using the unpaired t test where *** indicates p < 0.001 compared to untreated sample; and # indicates p < 0.05 compared to the sample without CVF.

Given the RNA degrading, membrane permeant, and protein unfolding properties of LA [64,65,68–71] it is possible that LA treatment of HIV-1 may lead to degradation of viral genomic RNA packaged within the viral core that would potentiate a decrease in HIV-1 infectivity. To address this question HIV_RHPA_ was treated with an equivalent 20 mM concentration of protonated L-, D- and DL-LA or acetic acid at pH 4.2 together with a low pH control (HCl, pH 4.2) for 5 min, neutralised and viral RNA extracted and quantified by qRT-PCR. We observed a significant decrease in HIV-1 RNA indicating degradation of HIV-1 genomic RNA following treatment with L-, D- and DL-LA, which all mediated an approximately 50% reduction in levels of amplifiable HIV-1 RNA as compared to untreated virus (p < 0.001 for all forms of LA; Fig 6A). No significant reduction in HIV-1 RNA levels was detected following treatment with an equimolar concentration (20 mM) of protonated acetic acid at the same pH (4.2) or low pH alone (HCl).

To determine if LA treatment promotes degradation of virion-associated HIV-1 RNA within the context of the cervicovaginal environment in which it is produced, the above analyses were repeated in the presence of pooled CVF derived from women with an optimal vaginal microbiota. Under these conditions, DL-LA treatment not only retained significant viral RNA degrading ability, but the extent of RNA degradation was enhanced in the presence of CVF (p = 0.03 vs no CVF condition; Fig 6B). This enhancement may be due to the presence of endogenous LA and/or other bacterial components in CVF from women with a *Lactobacillus*-dominated microbiota that increase viral permeability following LA treatment, which facilitates penetration of virucidal factors within CVF such as proteases and nucleases. Treatment of HIV-1 with acetic acid at pH 4.2, low pH 4.2 (HCl) alone or sodium lactate at pH 7 (osmolality alone) did not significantly affect HIV-1 RNA levels in the absence or presence of CVF (Fig 6B). Taken together, these data indicate that LA treatment has a specific ability to promote rapid degradation of HIV-1 viral RNA compared to other acids by penetrating the virion lipid envelope and core that contributes its potent inhibition of HIV-1 infectivity.

### LA inactivates both HIV-1 and HSV-2, but through different mechanisms

**Fig 7:**
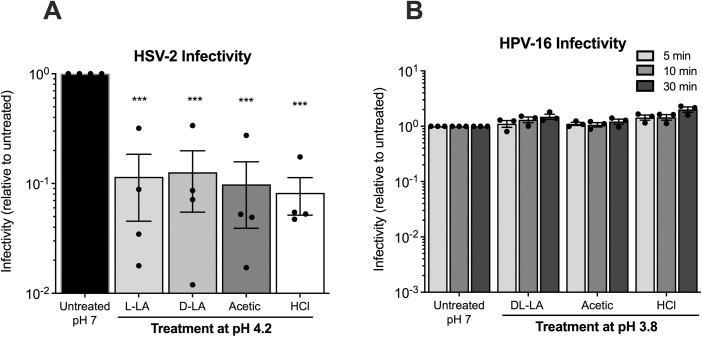
Virucidal activity of vaginal acids against HSV-2 and HPV-16. (A) HSV-2 was treated with 33 mM protonated D-LA, L-LA, DL-LA, acetic acid or low pH control (all at pH 4.2) at 37°C for 5 min and infectivity of neutralised virus assessed by a plaque assay in Vero cells. (B) HPV-16 pseudovirus was treated with 54 mM protonated DL-LA, acetic acid or low pH control (all at pH 3.8) at 37°C for the indicated times and HPV-16 infectivity assessed by luciferase assay. Graphs show mean ± SEM relative to untreated virus from at least n = 3 independent assays, represented by solid black circles. Statistical significance was assessed using the unpaired t test where *** indicates p < 0.001 compared to the untreated control.

In addition to HIV-1 and HIV-2 [40], LA has potent virucidal activity against HSV-1 and HSV-2 at low pH [47,48], although it is not clear whether the effects against these viral STIs, which also have a lipid envelope, are mediated by the same mechanism. To address this question, HSV-2 was treated with 0.3% (w/w) (33 mM) L-LA, D-LA and DL-LA or acetic acid at pH 4.2 or a low pH (4.2) control (HCl). Assays were performed at pH 4.2, rather than lower pH (i.e. pH 3.8), to maximise observing any differences in the virucidal activity of the acids against HSV-2. All samples were treated with acid for 5 min at 37°C, neutralised, and HSV-2 infectivity determined using a plaque assay. All acids, including the low pH (HCl) control, mediated a significant and equivalent reduction in HSV-2 infectivity (Fig 7A, p < 0.001 for all), indicating inactivation is likely due to a low pH environment and not due to any acid-specific activity.

To determine whether LA was virucidal against non-enveloped STI viruses we evaluated its virucidal activity against pseudoviruses, representing the non-enveloped human papillomavirus type 16 (HPV-16). To maximise observing a virucidal effect we treated HPV-16 with a higher physiological concentration of DL-LA and acetic acid, as well as HCl, all at a lower pH (3.8) for 5, 10 and 30 min at 37^o^C. Viral infectivity of neutralised samples was assessed using a luciferase-based infectivity assay. We observed no virucidal activity of DL-LA, acetic acid or low pH 3.8 alone against the HPV-16 pseudovirus (Fig 7B). Overall, these data indicate that HSV-2 inactivation is likely mediated primarily by low pH whereas HPV-16 pseudovirus is not inactivated by low pH or vaginal acids, suggesting that the LA virucidal activity against HIV-1 is virus-specific.

## Discussion

This study is the first to show the distinct and potent HIV-1 virucidal activity of LA compared to SCFAs and succinic acid present at physiological concentrations and pH that may be encountered by HIV-1 either shed or deposited in the vagina of women with an optimal vaginal microbiota compared to women with BV. Our study has defined mechanisms by which LA inactivates HIV-1 at the molecular level. We demonstrate that LA’s previously reported irreversible and potent HIV-1 virucidal activity [40] is not simply due to virion lysis or loss of surface gp120, but rather through multiple effects. These include targeting protein and nucleic acid packaged within the virion likely mediated by the membrane permeant, protein unfolding, and RNA degradation properties of the biologically active protonated form of LA [64,65,68–71] leading to inhibition of virion-associated HIV-1 RT activity and degradation of viral genomic RNA. The inhibitory effects of LA treatment on HIV-1 RT and viral RNA were significantly more potent than HCl or acetic acid, the smallest carboxylic acid elevated in vaginal fluid in women with BV, indicating an LA-specific effect. We also demonstrate that while LA has virucidal activity against HSV-2, another viral STI with a lipid envelope, this was a low pH effect, as similar inhibition was observed with other acids. Finally, we show that LA does not affect the infectivity of HPV-16, which lacks a lipid envelope. These findings extend our initial studies reporting the superior and potent virucidal activity of LA against a range of clinically relevant HIV-1 strains and HIV-2 compared to acetic acid, or low pH (i.e. HCl) [40] and highlight the important role of LA, compared to other carboxylic acid metabolites found in vaginal fluid, in attenuating the risk of a women acquiring and transmitting viral STIs.

In the current study we show that LA was responsible for mediating potent HIV-1 virucidal activity against a subtype B transmitted/founder strain, isolated from a female subject [40], in the context of a mixture of carboxylic acids at pH 3.8 observed in women with an optimal vaginal microbiota. In contrast, we showed that a carboxylic acid mixture and conditions simulating BV (at pH 5.0), lacked HIV-1 virucidal activity. This was observed despite there being an overall greater concentration of carboxylic acid metabolites (144 mM) in the mixture representing BV conditions relative to the acid mixture for women with an optimal vaginal microbiota (107 mM) [35]. Furthermore, the superior HIV-1 virucidal activity of LA compared to the other vaginal carboxylic acids was demonstrated under stringent conditions where LA, SCFAs (acetic, propionic and butyric acid) and succinic acid were each tested at equivalent concentrations of the protonated active form of the acid. Taken together, these data suggest that an optimal *Lactobacillus*-dominated vaginal microbiota, which produces LA, has the ability to inactivate HIV-1 present in the vaginal lumen. This is supported by an *ex vivo* study demonstrating the anti-HIV-1 activity of intrinsic LA in cervicovaginal secretions from women with *Lactobacillus*-dominated microbiota [39].

We performed studies to determine the HIV-1 virucidal mechanism of action of LA. We initially focused on effects of LA compared to HCl on the HIV-1 lipid membrane and the gp120 envelope protein [72], since they are both exposed on the outside of the virion. Distinct from a previous study that used a sucrose cushion to pellet virus [54] we used an OptiPrep gradient to separate viral particles from co-sedimenting microvesicles that may also contain viral proteins. We found that LA-treated HIV-1 particles remain intact, indicating that LA does not grossly disrupt the viral lipid membrane, and that the gp120 envelope remains virion-associated. Our data are consistent with the previous study reporting that LA treatment does not cause HIV-1 viral particle lysis [54].

While we observed that gp120 is present on the intact HIV-1 virion, the possibility remained that LA may have altered its conformation and function. Using SAXS analysis we found that treatment of recombinant monomeric HIV-1 gp120 with HCl (pH 4) results in rapid protein denaturation. In contrast, no global conformational changes were observed when gp120 was treated with either D-LA or L-LA at the same low pH found in women with an optimal vaginal microbiota, under conditions observed to dramatically decrease HIV-1 infectivity [40]. Our findings are partly consistent with a previous study that determined the conformational changes mediated by LA or HCl treatment on the protein, creatine kinase [65]. This study found that treatment with HCl resulted in aggregation of creatine kinase, similar to our findings with recombinant gp120. In contrast, treatment of creatine kinase with LA (at pH 3–4) elicited subtle protein unfolding in the absence of aggregation. Taken together this suggests that while we did not observe global changes in gp120 structure by SAXS, LA may still mediate changes in protein conformation, not detectable by SAXS, that could impact on its functions. Our analysis of the ability of LA-treated HIV-1 particles to bind to rCD4 *in vitro* demonstrated only a modest 2-fold decrease in binding; however, this saturated binding system may be less sensitive to subtle changes to CD4 binding affinity, which may have a much greater impact in the context of CD4 + T cell infection, where surface CD4 density is lower. It is also possible that LA may alter the conformation and function of trimeric virion-associated gp120 to inhibit binding to HIV coreceptors CCR4 or CCR5 and subsequent gp41 mediated viral fusion and entry [72]. In addition, the effect of LA on virion incorporated host proteins [e.g., leukocyte function-associated molecule 1 (LFA-1) or intercellular adhesion molecule (ICAM-1)], that promote HIV-1 binding to the cell surface [73,74], cannot be excluded. Despite these limitations our data indicate that any minor effects of LA on virion gp120 conformation, including its trimeric or protein secondary structure that mediates decreased rCD4-binding, is not LA specific and is unlikely to explain the potent HIV-1 virucidal activity mediated by LA.

Protonated LA is membrane permeant [64,68] and permeabilises the outer membrane of gram-negative bacteria [69,70]. Here we show that treatment of HIV-1 with protonated LA, but not the lactate anion, inhibits virion-associated RT activity requiring penetration of LA through the viral lipid envelope and core to directly target the RT. This LA treatment effect was more potent compared to equivalent levels of protonated acetic acid as well as media acidified to the same pH with HCl providing further evidence of the distinct abilities of these acids to penetrate the HIV-1 virion. Acetic acid is a smaller carboxylic acid compared to LA and has a higher acid dissociation constant (pKa 4.86) compared to 3.86 for LA, and accordingly at pH 3.8 there would be more protonated acetic acid compared to protonated LA. However, acetic acid is dramatically less efficient at killing BV-associated bacteria [44] as well as inactivating HIV-1 [40]. These findings indicate that LA may act through distinct mechanisms compared to acetic acid, including being more efficient at penetrating and/or subtly permeabilising the lipid envelope, penetrating the viral core and directly inhibiting RT. It is also possible that LA may alter the conformation and function of other critical viral structural proteins and enzymes

Levels of HIV-1 genomic RNA are typically quantified by qRT-PCR including determining viral load in the vagina of women with HIV [7,11–13]. Using qRT-PCR we show that LA treatment of HIV-1 particles results in degradation of the viral genomic RNA, which is potentiated in the presence of CVF. In contrast, we observed little effect on viral RNA degradation when HIV-1 was treated with acetic acid or HCl. The mechanism of this degradation may be due to either a direct degradation of viral RNA by LA, and/or an indirect effect. Supporting the former a previous study has shown that LA directly degrades nucleic acid including RNA purified from bacteria and mammalian cells [71]. HIV-1 genomic RNA is located within the viral core and is bound to viral nucleocapsid (NC). For LA to directly degrade the viral RNA genome it would need to penetrate the lipid envelope, viral core, and NC, which is possible due the membrane permeant and protein unfolding properties of LA [64,65,68–70]. Alternatively, LA, with a molecular weight of 90.08 Da, is considerably smaller than dNTPs (range 467 – 507 Da) and could potentially enter the core via its dynamic capsid pores that are normally used to import nucleotides required for reverse transcription [75]. Regarding an indirect effect on viral RNA degradation, the HIV-1 stock used here was in clarified conditioned media, from propagation in cell culture, which may contain factors from the host cell and/or media that contribute to viral RNA degradation and RT inactivation in LA-treated virions. These conditions may approximate *in vivo* conditions where LA acts to permeabilise (but not lyse) the virion and enable molecules in the conditioned medium and CVF, including proteases and ribonucleases, to inactivate HIV-1. The more potent degradation of HIV-1 genomic RNA when LA treatment was performed in CVF (Fig 6B), also supports contribution from an indirect effect. LA’s combined effects on virion-associated RT function and the viral RNA genome template required for generation of the proviral DNA precursor in infected cells likely explains the greater decrease in HIV-1 infectivity compared to treatment with the same molar concentration of protonated acetic acid (Fig 5B).

We extended our analysis to HSV-2, another enveloped viral STI, to determine if there were similarities between inactivation of HIV-1 and HSV-2 by LA compared to acetic acid and low pH alone (HCl). HSV-2 causes genital herpes and possesses a double-stranded DNA genome. In contrast to HIV-1, our data show that both the L- and D-LA isomers have HSV-2 virucidal activity that is similar in potency to acetic acid and acidity alone (pH 4.2, HCl adjusted). Our findings are consistent with a previous study reporting that the HSV-2 virucidal activity of LA and low pH alone are similar [48]. Thus, not all enveloped viral STIs are inactivated by LA and other acids in the same manner with the inactivation of HIV by LA being distinct and specific [40]. This may be related to differences in membrane composition, internal proteins and RNA as compared to DNA genomes of HIV-1 and HSV-2, respectively.

In contrast to HIV-1 and HSV-2 we found that the HPV-16 pseudovirus, representing a non-enveloped viral STI [76], was not inactivated by DL-LA, acetic acid, or pH 3.8 (HCl). While there are little data on the HPV virucidal activity of acids, nonenveloped viruses (i.e., rhinoviruses) are reported to be acid labile at a pH below 5.3 [77,78]. The lack of virucidal effect of LA on HPV-16 may be explained by the effect of low pH on HPV-16 PsV L1 and L2 capsid proteins, which undergo a maturation process during virus production [79]. This maturation stabilizes the HPV capsid, by reinforcing the intermolecular disulfide bonds between adjacent L1 molecules, forming pentamers, which is disrupted under alkaline conditions [79,80]. Thus, acidic conditions, such as those in our studies, would be expected to promote the stabilisation of the capsid [79,81].

The more potent HIV-1 virucidal activity of LA relative to low pH and acetic acid [40] is similar to other studies reporting LA’s microbicidal activity against BV-associated bacteria and *Neisseria gonorrhoeae* [44,45,82]. This suggests that LA, present at high concentrations in women with an optimal vaginal microbiota [36,56], acts to protect the lower FRT from reproductive tract pathogens to a greater extent than acetic acid, which predominates during BV [83,84]. It also confirms that LA has inherent HIV-1 virucidal activity that is not simply a low pH effect [44,45,82]. These findings indicate that the metabolite shift in the lower FRT that occurs during BV may increase HIV risk in several ways. These include loss of virucidal and bactericidal levels of LA and its replacement with SCFAs and succinic acid that do not target HIV-1, or other viral and bacterial STIs as well as BV-associated bacteria known to promote increased HIV acquisition and transmission [13].

The loss of virucidal activity of vaginal microbiota carboxylic acid metabolites during BV has important implications for HIV transmission, particularly in the context of mother-to-child transmission during vaginal birth and potentially during female-to-male transmission and male-to-female transmission [40]. Vaginal HIV load is greater in the presence of BV, which is associated with an increased risk of HIV transmission to a male partner or neonatal child of a women with HIV [7,13,85,86]. The potent virucidal activity of LA, which we have shown can be mediated *in vitro* in the presence of CVF from women with and without BV, would be anticipated to directly inactivate HIV shed into the vagina of women with HIV, and indeed the presence of vaginal lactobacilli negatively correlates with viral load in vaginal fluid [7,11,13,87,88]. This notion is supported by our previous study showing that native LA present in CVF from women with a *Lactobacillus-*dominated microbiota potently inactivates HIV-1 *ex vivo* [39].

In contrast, our findings show that BV-associated SCFAs have no observable virucidal activity under conditions that prevail during BV. This could explain higher viral load observed in women with HIV who also have BV and increased transmissibility to their male partners [13]. Additionally, LA may indirectly impact HIV, by suppressing growth of BV-associated bacteria [44] and preventing BV and cervicovaginal inflammation, which is associated with an increased risk of HIV acquisition in both males and females [13,23]. Furthermore, the virucidal activity of LA may synergise with the immunomodulatory effects of LA [41] and vaginal lactobacilli [89–91] to suppress inflammation-related HIV target cell activation (e.g., from resident memory CD4 + T cell reservoirs) [92], and recruitment [93,94], to further protect from HIV transmission.

The findings on the potent antiviral activity of LA against HIV and other viral and bacterial STIs, along with its direct anti-inflammatory [41,42] and epithelial barrier integrity strengthening effects on cervicovaginal epithelial cells [34] *in vitro* and *ex vivo* need to be confirmed in well-designed clinical studies in women [95]. However, LA could potentially be advanced as an adjunct to antibiotics and/or antiretrovirals to optimise the vaginal microbiota to prevent women acquiring as well as transmitting HIV to their babies and partners. LA could be delivered directly by gel or by sustained intravaginal delivery to decrease infectious HIV shed into the vaginal lumen in pregnant women living with HIV. Alternatively, women with BV could be treated with LA to optimise their vaginal microbiota by fostering colonisation with beneficial *Lactobacillus* spp. that produce LA. Standard of care for BV is treatment with antibiotics, which while resulting in short-term cure, has a high recurrence rate (58% within 12 months) [96], if the male partner is not concurrently treated with antibiotics [97], and does not promote the presence of a stable and non-inflammatory *L. crispatus*-dominated vaginal microbiota [98]. Novel strategies are being employed to optimise the vaginal microbiota including the advancement of vaginally-derived *Lactobacillus* spp. as therapeutics. In this regard, Lactin V, a vaginally applied *L. crispatus*-based live biotherapeutic that produces LA, has been shown to prevent BV recurrence in 30% of cases compared to placebo following metronidazole therapy in a phase 2 randomized placebo-controlled trial [99] as well as decreasing genital inflammation [100]. Other investigators are pursuing vaginal microbiome transplants or the use of combinations of more than one *L. crispatus* strain to maximise vaginal colonisation [6,101,102]. Given the link between BV and many adverse health outcomes beyond HIV acquisition including other STIs, preterm birth, pelvic inflammatory disease, endometritis, cervical dysplasia, and infertility, if successful, these strategies are anticipated to have a major impact on a women’s sexual and reproductive health.

## Materials and methods

### Ethics statement

Ethical approval for collection of cervical vaginal fluid was obtained from Homewood Institutional Review Board, Johns Hopkins University HIRB00000526, and the Alfred Human Ethics Research Committee, Project Numbers 80/13 and 238/23. Formal written consent was obtained from participants who provided CVF samples.

### Cell culture and virus production

The HIV-1 permissive TZM-bl reporter cell line [103] and 293T cells were obtained through the NIH HIV Reagent Program and cultured in Dulbecco’s Modified Eagle’s Medium (DMEM; ThermoFisher, Waltham, MA) supplemented with 10% (v/v) heat-inactivated fetal calf serum (FCS; Sigma-Aldrich, St Louis, MO), 100 U/mL penicillin, 100 µg/mL streptomycin, and 2 mM L-glutamine (all from ThermoFisher; DMEM-10). Phytohaemagglutinin (PHA)-stimulated human peripheral blood mononuclear cells (PBMCs) from HIV seronegative donors were prepared as previously described [40] prior to infection with HIV. Infectious HIV-1 subtype B transmitted/founder strain, HIV_RHPA_, was generated from the molecular clone pRHPA.c/2635 (HIV Reagent Program) by calcium phosphate transfection of 239T cells followed by propagation in human PBMCs as described previously [40]. The HIV-1 subtype B CCR5-coreceptor using HIV_BaL_ was propagated by infection of PHA-stimulated PBMCs and purified by ultracentrifugation through a sucrose cushion as previously described [39,40].

### Treatment of HIV-1 with vaginal acids

DL-LA was prepared from an 85% (w/w) solution (Sigma-Aldrich), D-LA was prepared from D-(-)-LA crystalline powder (Chem-Impex International Wood Dale, IL, USA), L-LA was prepared from a 30% (w/w) L-(+)-LA solution (Sigma-Aldrich), acetic acid was prepared from 99.5% (w/w) glacial acetic acid (Merck, Kenilworth, NJ), and sodium lactate was prepared from ~98% crystalline powder (Sigma-Aldrich). Solutions were pH-adjusted using hydrochloric acid (HCl) or sodium hydroxide (Sigma-Aldrich). All acids were of American Chemical Society (ACS) or reagent grade. All stock solutions were volumetrically prepared to 1 M stock solutions.

To simulate vaginal carboxylic acid composition associated with an optimal microbiota, HIV-1 virions were treated in DMEM-10 containing DL-lactic acid alone (100 mM, adjusted to pH 3.8) or a mixture (DL-LA + optimal SCFA) of 100 mM lactic acid, 4 mM acetic acid, 1 mM propionic acid, 1 mM butyric acid, and 1 mM succinic acid (adjusted to pH 3.8) to reflect levels of LA, SCFA and succinic acid associated with a *Lactobacillus*-dominated microbiota (S1 Table) [35]. To simulate vaginal acid composition associated with BV, HIV-1 virions were treated with acetic acid (100 mM, pH 5.0) or a mixture (Non-optimal SCFA) of acids comprising of 20 mM DL-lactic acid, 100 mM acetic acid, 2 mM propionic acid, 2 mM butyric acid, and 20 mM succinic acid (adjusted to pH 5.0) (S1 Table). DMEM-10 adjusted to the corresponding pH with HCl alone was included in all experiments as a control. Virus was incubated for 5, 10, and 30 min at 37°C with continuous gentle stirring. At each time-point 200 μL aliquots of treated virus were removed and immediately neutralised by 10-fold dilution in DMEM-10 containing 25 mM HEPES [4-(2-hydroxyethyl)-1-piperazineethanesulfonic acid]. This method was used to neutralise LA in subsequent experiments, unless otherwise stated. Viral infectivity was determined in the TZM-bl reporter cell line using the β-galactosidase infectivity assay to quantify blue-stained HIV-infected cells as previously described [40].

To directly compare virucidal activity between acids with different acid dissociation constants (i.e., LA pK_a_ 3.86, acetic acid pK_a_ 4.76, propionic acid pK_a_ 4.88, butyric acid pK_a_ 4.83, succinic acid pK_a_ 4.16 and 5.61), HIV_RHPA_ was treated at 37°C with an equimolar 20 mM concentration of protonated vaginal carboxylic acids, calculated for a specific pH of 4.2 using the Henderson-Hasselbalch equation: % Dissociation = 100/(1 + 10^(-1(pH-pKa)^) [36]. Since the anion concentration contributes to the osmolality of the treatment solution and varies for each carboxylic acid, sodium lactate was employed as an osmolality control (~473 mOsm/kg) where osmolality was greater than the highest osmolality for any of the acid treatments (∼357 mOsm/kg). HIV-1 virucidal activity of the acids and viral infectivity were determined as above.

### Iodixanol velocity gradient purification of lactic acid treated HIV-1

Iodixanol (OptiPrep) solutions (Axis-Shield, Oslo, Norway) were prepared by diluting Optiprep (60% w/v iodixanol in water) with Mg^2+^ and Ca^2+^ free phosphate buffered saline (PBS-) to give four density solutions of 6%, 10%, 14% and 18% (w/v) which were overlayed from most to least dense and allowed to diffuse overnight at 4°C to generate a linear gradient, according to the manufacturer’s recommendations. Virus samples were ultracentrifuged at 250,000 x *g* for 1.5 h at 4°C, and five 2 ml fractions were collected and the top two and bottom three fractions were pooled, the latter containing HIV-1 in the absence of contaminating microvesicles [61], and treated with 0.1% Triton-X100 for Western blot analysis. Viral proteins were precipitated as previously described [104], diluted in sodium dodecyl sulfate (SDS) loading dye (6.25 mM Tris pH 6.8, 1% w/v SDS, 0.01% v/v bromophenol blue, 10% glycerol, 0.3 M β-mercaptoethanol) and heated for 10 min at 95^o^C prior to separation by SDS-polyacrylamide gel electrophoresis (SDS-PAGE). Separated proteins were transferred to a Hybond nitrocellulose membrane (GE Healthcare, Chicago, IL), blocked with 2.5% skim-milk powder in PBS- and HIV-1 proteins detected with HIV-1 positive serum provided by Dale McPhee (Burnet Institute, Melbourne, VIC, Australia) and secondary goat anti-human IgG antibody conjugated to IRDye 800 (Invitrogen, Waltham, MA). Membranes were scanned with the Licor Odyssey system (LICOR, Lincoln, NE) and quantified using the Image Studio Lite software.

### Small angle X-ray Scattering (SAXS) analysis

Soluble monomeric gp120 glycoprotein derived from the subtype B CCR5-utilising strain NL(AD8) [105] was obtained from media conditioned by a stably transfected HeLa clone expressing cleavable gp140 [106]. Purification was performed using Lentil Lectin affinity chromatography followed by size exclusion chromatography [107]. Purified gp120 protein (0.1-0.2 mg/ml) in PBS- was treated with a concentration series of L-LA or D-LA at a pH found in women with an optimal vaginal microbiota (close to the pKa of LA) from 0.01% to 1% (w/w) at pH 4 for 10–15 min at room temperature. We used pH 4 in these experiments since we have previously shown that treatment of HIV-1 with LA at pH 4 results in >10,000-fold decrease in HIV-1 infectivity compared to untreated virus [40]. LA treatments were compared with untreated gp120 protein at pH 7 and gp120 treated with pH 4 (PBS- acidified with HCl). A no protein control was also included as reference. Analyses were performed at the SAXS/WAXS beamline, Australian Synchrotron. Parameters for SAXS data collection [63] are described in S2 Table. Briefly, samples were flowed at 1 μl/s during data collection and 10 x 1 second exposures were measured to control for radiation damage. Raw scattering images were averaged, and the appropriate reference data was subtracted from each corresponding gp120 sample using the same conditions. ScatterBrain IDL software (Australian Synchrotron) was used for averaging and reference subtraction, and data was analysed using PRIMUS from the ATSAS package [108].

### CD4 binding ELISA

HIV_BaL_ viral supernatant was treated with 1% (w/w) L-LA at pH 3.8, acidified media at pH 3.8 (HCl adjusted) or sodium L-lactate at neutral pH for 60 min at 37°C then binding to CD4 assessed using an in-house CD4 binding ELISA. Plates coated with mouse anti-CD4 antibody (4B4 clone) were incubated with 80 ng of recombinant soluble CD4 (rCD4) engineered with a deletion in the transmembrane region but retaining the cytoplasmic tail (a gift from Nadine Barnes and David Anderson, Burnet Institute) reconstituted in lysis buffer (10% Triton X100, and 1 µg/ml each of leupeptin, aprotinin and pepstatin A in PBS-) for 1 h at room temperature. Excess rCD4 was removed by washing six times with wash buffer (0.05% Tween-20 in PBS-; 300 μl/well per wash). Treated virus solutions (100 μl/well) were added to plates, incubated for 1 h at room temperature and wells subsequently washed three times with wash buffer and three times with PBS-. Bound virus was quantified using the RETRO-TEK HIV-1 antigen ELISA kit (ZeptoMetrix Corporation, Buffalo, NY) according to the manufacturer’s instructions. Viral infectivity was determined in parallel in the TZM-bl cell line as above.

### Analysis of virion-associated RT activity and recombinant RT activity

The reverse transcriptase (RT) assay was used to detect RNA dependent DNA polymerase (RDDP) activity of virion-associated RT from PBMC-derived HIV_RHPA_ supernatants and recombinant RT treated with acids, followed by neutralisation. These assays use an exogenous RNA/DNA template/primer to measure RDDP activity. For quantitation of virion-associated RT activity using a radiolabelled substrate, neutralised viral supernatants were lysed with an equal volume of 0.3% IGEPAL CA-630 (Sigma-Aldrich) and then added to the RT reaction mix containing a final concentration of 5 µg/ml poly(rA)/oligo(dT) template/primer, 10 µCi [α-^33^P]-deoxythymidine triphosphate (dTTP) (PerkinElmer, Waltham, MA, USA), 50 mM Tris pH 8, 7.5 mM KCl, 2 mM dithiothreitol and 5 mM MgCl_2_ for 1 h at 37^o^C as published [109]. The reaction was stopped by applying samples to Whatman DE81 anion exchange paper (Sigma-Aldrich), followed by washing in 2x SSC buffer (300 mM sodium chloride, 30 mM sodium citrate), then 95% ethanol and air dried. The incorporated radiolabel was quantified using the Typhoon Trio Phosphorimager (GE Healthcare, Little Chalfont, BUX, UK) and Image Quant software (GE Healthcare). The average signal for the background control, containing media only, was subtracted from quantified samples. For assays using recombinant HIV-1 RT (subtype B), the p66/p51 HIV-1 RT heterodimer, engineered with an N-terminal histidine tag, was expressed and purified from the p6HRT-PR vector (kindly provided by Nicolas Sluis-Cremer) as published [66]. RDDP assays were performed as above except in the presence of recombinant HIV-1 RT (25 – 100 ng) where RT treated with acids was neutralised before adding to the reaction mix.

Virion-associated RT activity following acid treatment in the presence of CVF was quantified using a non-radioactive product-enhanced reverse transcriptase (PERT) assay [67]. Immediately following acid treatment, HIV-1 virions were disrupted in 2x PERT lysis buffer [100 mM Tris pH 7.4, 50 mM KCl, 0.25% (v/v) Triton X-100, 40% (v/v) glycerol]. RT activity in lysates was quantified by qPCR of cDNA synthesised from an MS2 RNA (40 nM) template (Roche) in a reaction mix containing Agilent Brilliant III Ultra-Fast SYBR Green low ROX qPCR Master Mix (Agilent), RNAsin ribonuclease inhibitor (Promega), and 200 nM of MS2 forward (5-TCCTGCTCAACTTCCTGTCGAG-3) and reverse (5-CACAGGTCAAACCTCCTAGGAATG-3) primers. PERT was performed using the QuantStudio 7 Flex with 20 min incubation at 42^o^C for cDNA synthesis by virion-associated RT followed by incubation for 5 min at 40^o^C, to activate the Hot-start Taq enzyme, followed by 40 cycles at 95°C for 5 seconds and 60°C for 60 seconds. A standard curve was generated for each assay using recombinant M-MuLV reverse transcriptase (New England Biolabs) to quantify virion-associated RT activity in samples.

### Collection and processing of CVF

Cervicovaginal fluid (CVF) was collected from women of reproductive age (18–45 years old) with optimal *Lactobacillus*-dominated microbiota (Nugent score 0–3) recruited at the Johns Hopkins University Campus using a menstrual SoftCup (Instead Inc., La Jolla, CA) as previously described [110]. CVF samples from three women were pooled, and the pH of the pooled CVF was 3.83. CVF samples were also collected using the SoftCup from women of reproductive age attending the Melbourne Sexual Health Centre and processed as previously described [110]. BV was diagnosed using the Nugent score [111]. For *in vitro* experiments, CVF samples were pooled from two women who were BV negative, both with Nugent scores of 0 and vaginal pH 4.0, and two women who were BV positive, both with Nugent scores of 8 and vaginal pH 5.0.

### Acid treatment in the presence of CVF

Sixty microlitres of pooled CVF was prepared and added to an equal volume of HIV_RHPA_ in culture medium, with the virus propagated in PBMCs as described above. Mixtures of CVF and virus were adjusted to the required final pH and LA concentrations and incubated for 5 min at 37^o^C with continuous mixing. Immediately following incubation aliquots were processed for determination of infectivity, virion-associated RT activity, and quantitation of viral RNA. For virion-associated RT activity, an aliquot was immediately removed after incubation and added to 2 x PERT lysis buffer. To quantify infectivity and virion-associated RNA, treated samples were immediately neutralised by 10-fold dilution in DMEM-10 containing 25 mM HEPES.

### Analysis of virion-associated RNA following treatment with carboxylic acids

Following acid treatment of HIV-1, viral RNA was extracted from neutralised supernatant using the QIAamp Viral RNA Extraction Kit, according to manufacturer’s instructions (QIAGEN, Hilden, Germany). Contaminating DNA was removed by treatment with RNase free DNase I and heat inactivated according to the manufacturer’s instructions (Roche, Basel, Switzerland). Complementary DNA was synthesized using the Transcriptor First Strand cDNA Synthesis Kit (Roche) and subjected to quantitative Reverse Transcriptase Polymerase Chain Reaction (qRT-PCR) using the Brilliant II SYBR qPCR Master Mix (Agilent Technologies, Santa Clara, CA) containing 300 nM of each primer targeting the HIV-1 R-U5 region: hRU5-F2 5’-GCCTCAATAAAGCTTGCCTTGA-3’ and hRU5-R 5’-TGACTAAAAGGGTCTGAGGGATCT-3’ [112]. Cycling conditions were 95°C for 10 min, followed by 40 cycles of 95°C for 30 sec and 60°C for 1 min with fluorescence detected using the Mx3005P instrument (Agilent). Absolute DNA copies were calculated using a standard curve derived from serial dilutions of plasmid DNA as previously [113].

### Production and infectivity of HSV-2

Herpes simplex type 2 (HSV-2) stocks were generated by infection of Vero cells (ATCC CCL-81) cultured in DMEM with 3% FCS (DMEM-3) with the HSV-2 G strain (ATCC item number VR-734) as previously described [114]. Infected cells and supernatants were collected and frozen at -80°C for >1 h, with subsequent thawing and re-freezing for a total of three times to ensure HSV-2 release from cells. Viral supernatant was clarified by low-speed centrifugation and stored at -80°C. HSV-2 infectivity was determined by plaque assay in Vero cells, where cells were infected for 3–4 h, viral inoculum removed and cells overlayed with 1.6% low viscosity carboxymethyl cellulose (Sigma-Aldrich) prepared in DMEM-3. After incubation for 48 h, cells were fixed with methanol, washed with PBS- then stained with 0.1% Crystal violet, and plaque forming units/ml counted manually using light microscopy.

### Production and infectivity of human papilloma pseudovirus

Human papillomavirus type 16 (HPV 16) pseudoviruses (PsV) were generated by transfection of 239TT cells with p16sheLL and pCLucf (a gift from J. T Schiller) expressing HPV capsid proteins and the luciferase reporter pseudogenome, respectively as previously described [115]. Transfected cells were pelleted and resuspended in PBS- containing 9.5 mM MgCl_2_, 4% Triton X-100, RNase A (7 U/mL) and 25 mM ammonium sulfate [79]. Cell lysate was incubated at 37°C for 20–24 h to allow pseudovirus maturation, clarified by centrifugation, and the supernatant containing mature HPV PsV was transferred to siliconised tubes for storage at -80°C.

### Statistical analyses

Statistical significance for virucidal, rCD4 binding, RT activity, qRT-PCR to detect viral genomic RNA, and Western blot analyses were performed using either an unpaired or paired t test as indicated in the text using GraphPad PRISM version 10.1.0 software. All data were derived from at least n = 3 independent assays unless otherwise stated in the Figure legend.

## Supporting information

S1 Fig Anti-HIV-1 activity of carboxylic acids at neutral pH. HIV_RHPA_ was treated for 30 min at 37^o^C with 100 mM of each carboxylic acid. Viral infectivity relative to the untreated control (untreated) was determined in the TZM-bl reporter cell line. Error bars denote the mean ± SD from two independent experiments represented by solid black circles.
(PDF)

S2 Fig Relative infectivity of HIV-1 treated with L-LA at pH 3.8 compared to untreated virus (UT). Infectivity was determined from the same treated samples tested in parallel as described in Fig 3. (A) with 0.3% (w/w) (33 mM) L-LA in experiments to determine effect of L-LA on virion integrity or Fig 4C (B) with 1% (w/w) (110 mM) L-LA to determine ability of treated virions to bind to recombinant CD4. Error bars denote the mean ± SEM from n = 3 independent experiments represented by solid black circles.
(PDF)

S3 Fig Inhibition of virion-associated reverse transcriptase (RT) activity and HIV-1 infectivity by DL-LA in the presence of cervicovaginal fluid (CVF) from women with and without bacterial vaginosis (BV). HIV_RHPA_ treated with 20 mM of protonated DL-LA (37 mM DL-LA) at pH 3.8 in the presence or absence of an equal volume of pooled neat cervicovaginal fluid (CVF) from women with bacterial vaginosis (CVF BV+) or women without BV (CVF BV-), or treatment at pH 3.8 (HCl) or with sodium-lactate at pH 7 (Na-Lactate pH 7) for 5 min at 37^o^C compared to untreated virus (untreated pH 7). (A) Virion-associated RT activity compared to untreated virus as determined using the product-enhanced reverse transcriptase (PERT) assay. Aliquots were lysed in PERT lysis buffer immediately following incubation before being subjected to PERT. (B) HIV_RHPA_ infectivity following treatment as determined in the TZM-bl infectivity assay. Immediately after incubation, samples were neutralised by dilution in DMEM-10 containing 20 mM HEPES before being assayed in the TZM-bl assay. Error bars denote the mean ± SEM from n = 4 independent experiments represented by solid black circles. Statistical significance was determined using the unpaired t test where ** and **** represent p = 0.0038 and p < 0.0001, respectively.
(PDF)

S4 Fig Activity of purified reverse transcriptase (RT) after acid treatment. Purified recombinant HIV-1 RT was incubated with the indicated treatments for 2 min at 37^o^C and RT activity determined after sample neutralisation using a radiolabelled ^33^PdTTP substrate. (A) RT treated with increasing concentrations of L-lactic acid (L-LA) compared to untreated control (UT). (B) RT treated with L-lactic acid, D-lactic acid (D-LA), racemic lactic acid (DL-LA), acetic acid and media acidified with HCl, all adjusted to pH 3.8. Error bars denote the mean ± SEM from n = 6 technical replicates from one assay.
(PDF)

S1 Table Lactic acid, short chain fatty acids, and succinic acid concentrations representing an optimal vaginal microbiota and BV. (PDF)

S2 Table SAXS data collection and scattering derived parameters. (PDF)

S1 File Data set. (XLSX)
