# Supplementary material for: Lactic acid produced by optimal vaginal Lactobacillus spp. potently and specifically inactivates HIV-1 in vitro by targeting the viral RNA genome and reverse transcriptase
Source: PLoS Pathog. 2025 Oct 10;21(10):e1013594. doi: 10.1371/journal.ppat.1013594 (PMC12527216; doi:10.1371/journal.ppat.1013594)
Supplement: S1 Fig — HIVRHPA was treated for 30 min at 37oC with 100 mM of each carboxylic acid. Viral infectivity relative to the untreated control (untreated) was determined in the TZM-bl reporter cell line. Error bars denote the mean ± SD from two independent experiments represented by solid black circles. (PDF) [file ppat.1013594.s001.pdf]

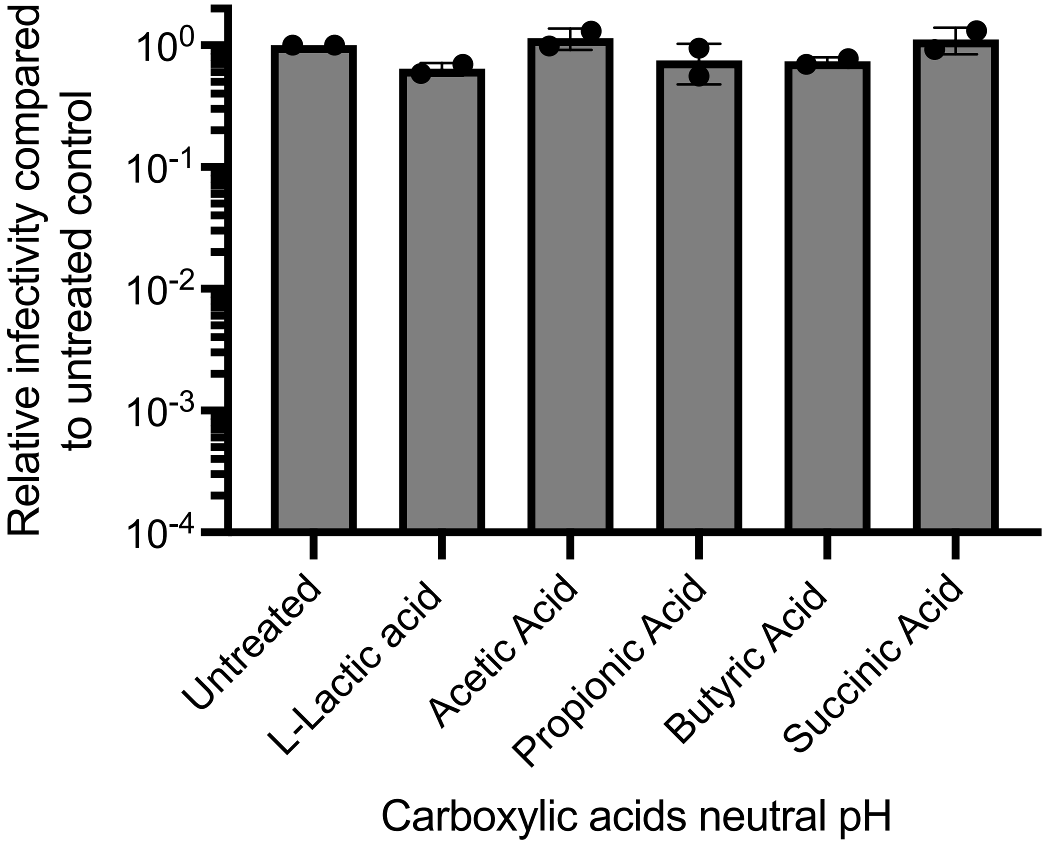

S1 Figure. Anti-HIV-1 activity of carboxylic acids at neutral pH. HIV_{RHPA} was treated for 30 min at 37°C with 100 mM of each carboxylic acid. Viral infectivity relative to the untreated control (untreated) was determined in the TZM-bl reporter cell line.

Error bars denote the mean ± SD from two independent experiments.
