# Supplementary material for: Lactic acid produced by optimal vaginal Lactobacillus spp. potently and specifically inactivates HIV-1 in vitro by targeting the viral RNA genome and reverse transcriptase
Source: PLoS Pathog. 2025 Oct 10;21(10):e1013594. doi: 10.1371/journal.ppat.1013594 (PMC12527216; doi:10.1371/journal.ppat.1013594)
Supplement: S2 Fig — Infectivity was determined from the same treated samples tested in parallel as described in Fig 3. (A) with 0.3% (w/w) (33 mM) L-LA in experiments to determine effect of L-LA on virion integrity or Fig 4C (B) with 1% (w/w) (110 mM) L-LA to determine ability of treated virions to bind to recombinant CD4. Error bars denote the mean ± SEM from n = 3 independent experiments represented by solid black circles. (PDF) [file ppat.1013594.s002.pdf]

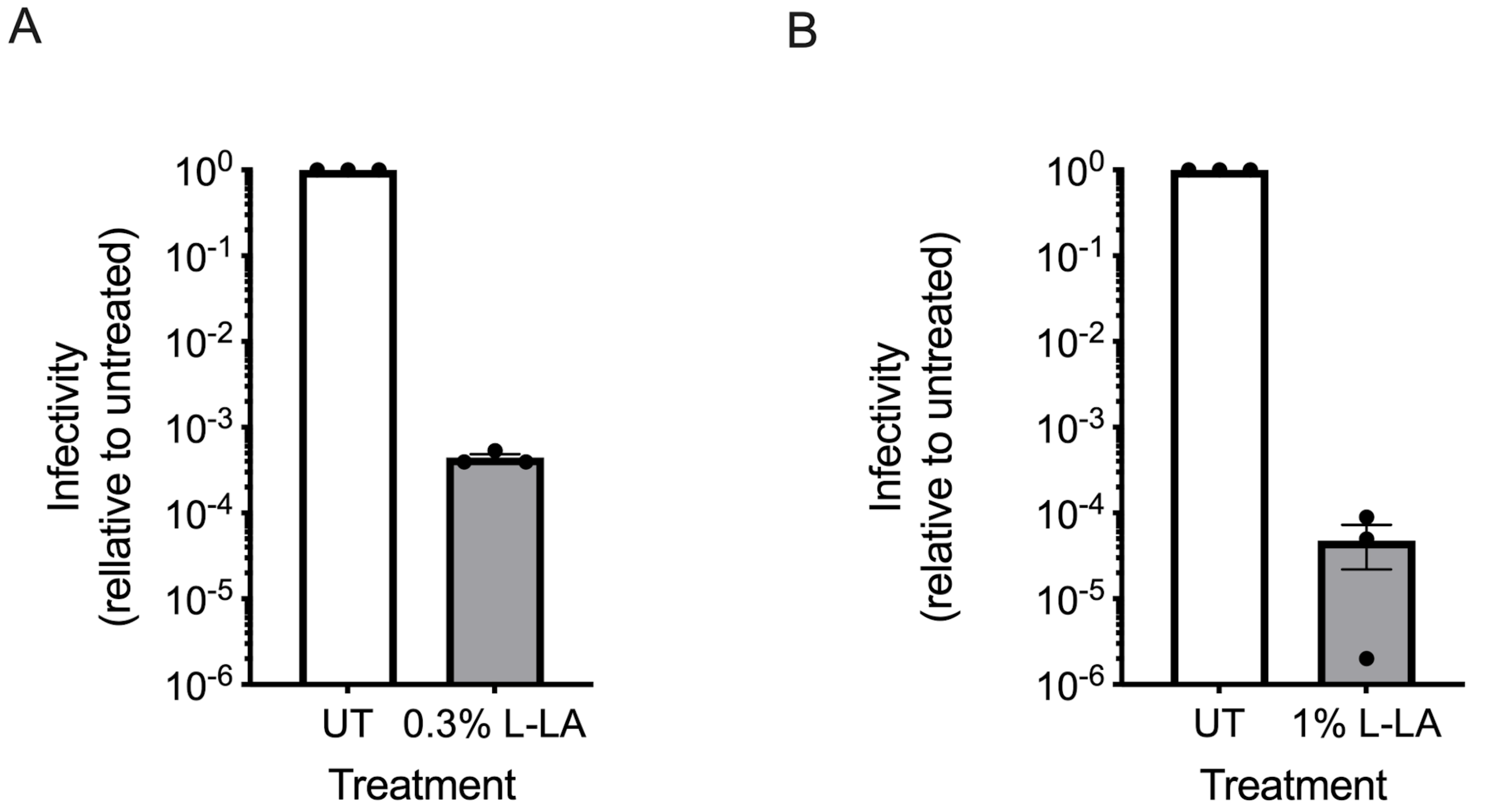

S2 Figure. Relative infectivity of HIV-1 treated with L-LA at pH 3.8 compared to untreated virus (UT). Infectivity was determined from the same treated samples tested in parallel as described in Fig 3. **(A)** with 0.3% w/w (33 mM) L-LA in experiments to determine effect of L-LA on virion integrity or Figure 4C **(B)** with 1 % w/w (110 mM) L-LA to determine ability of treated virions to bind to recombinant CD4. Error bars denote the mean ± SEM from n=3 independent experiments represented by solid black circles.
