# Supplementary material for: Lactic acid produced by optimal vaginal Lactobacillus spp. potently and specifically inactivates HIV-1 in vitro by targeting the viral RNA genome and reverse transcriptase
Source: PLoS Pathog. 2025 Oct 10;21(10):e1013594. doi: 10.1371/journal.ppat.1013594 (PMC12527216; doi:10.1371/journal.ppat.1013594)
Supplement: S3 Fig — HIVRHPA treated with 20 mM of protonated DL-LA (37 mM DL-LA) at pH 3.8 in the presence or absence of an equal volume of pooled neat cervicovaginal fluid (CVF) from women with bacterial vaginosis (CVF BV+) or women without BV (CVF BV-), or treatment at pH 3.8 (HCl) or with sodium-lactate at pH 7 (Na-Lactate pH 7) for 5 min at 37oC compared to untreated virus (untreated pH 7). (A) Virion-associated RT activity compared to untreated virus as determined using the product-enhanced reverse transcriptase (PERT) assay. Aliquots were lysed in PERT lysis buffer immediately following incubation before being subjected to PERT. (B) HIVRHPA infectivity following treatment as determined in the TZM-bl infectivity assay. Immediately after incubation, samples were neutralised by dilution in DMEM-10 containing 20 mM HEPES before being assayed in the TZM-bl assay. Error bars denote the mean ± SEM from n = 4 independent experiments represented by solid black circles. Statistical significance was determined using the unpaired t test where ** and **** represent p = 0.0038 and p < 0.0001, respectively. (PDF) [file ppat.1013594.s003.pdf]

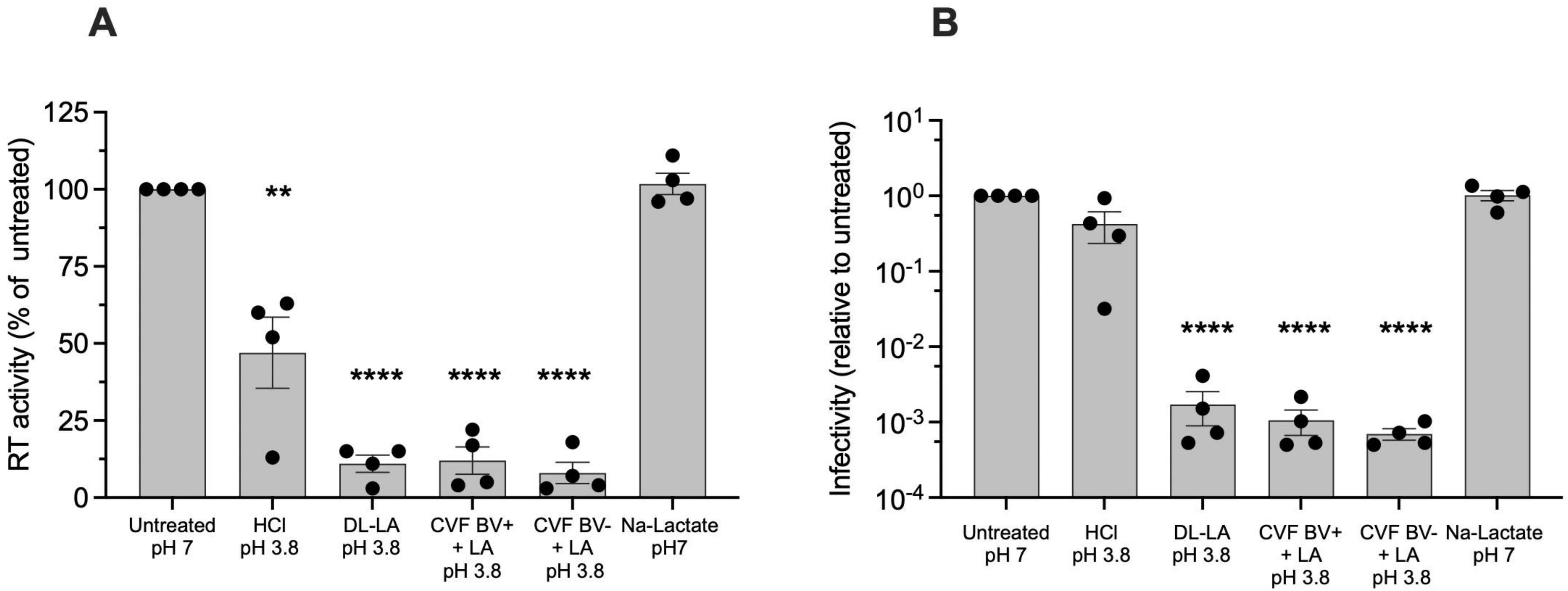

S3 Figure. Inhibition of virion-associated reverse transcriptase (RT) activity and HIV-1 infectivity by DL-LA in the presence of cervicovaginal fluid (CVF) from women with and without bacterial vaginosis (BV). HIV_{RHPA} treated with 20 mM of protonated DL-LA (37 mM DL-LA) at pH 3.8 for 5 min at 37°C in the presence or absence of an equal volume of pooled neat cervicovaginal fluid (CVF) from women with bacterial vaginosis (CVF BV+) or women without BV (CVF BV-), samples acidified to pH 3.8 with HCl alone or treatment with sodium-lactate at pH 7 (Na-Lactate pH 7) compared to untreated virus (Untreated pH 7). **(A)** Virion-associated RT activity compared to untreated virus as determined using the product-enhanced reverse transcriptase (PERT) assay. Aliquots were lysed immediately following incubation in PERT lysis buffer before being subjected to PERT. **(B)** HIV_{RHPA} infectivity following treatment as determined in the TZM-bl infectivity assay. Immediately after incubation, samples were neutralised by dilution in DMEM-10 containing 20 mM HEPES before being assayed in the TZM-bl assay. Error bars denote the mean ± SEM from n=4 independent experiments represented by solid black circles. Statistical significance was determined using the unpaired t test where ** and **** represent p= 0.0038 and p<0.0001, respectively.
