# Supplementary material for: Lactic acid produced by optimal vaginal Lactobacillus spp. potently and specifically inactivates HIV-1 in vitro by targeting the viral RNA genome and reverse transcriptase
Source: PLoS Pathog. 2025 Oct 10;21(10):e1013594. doi: 10.1371/journal.ppat.1013594 (PMC12527216; doi:10.1371/journal.ppat.1013594)
Supplement: S4 Fig — Purified recombinant HIV-1 RT was incubated with the indicated treatments for 2 min at 37oC and RT activity determined after sample neutralisation using a radiolabelled 33PdTTP substrate. (A) RT treated with increasing concentrations of L-lactic acid (L-LA) compared to untreated control (UT). (B) RT treated with L-lactic acid, D-lactic acid (D-LA), racemic lactic acid (DL-LA), acetic acid and media acidified with HCl, all adjusted to pH 3.8. Error bars denote the mean ± SEM from n = 6 technical replicates from one assay. (PDF) [file ppat.1013594.s004.pdf]

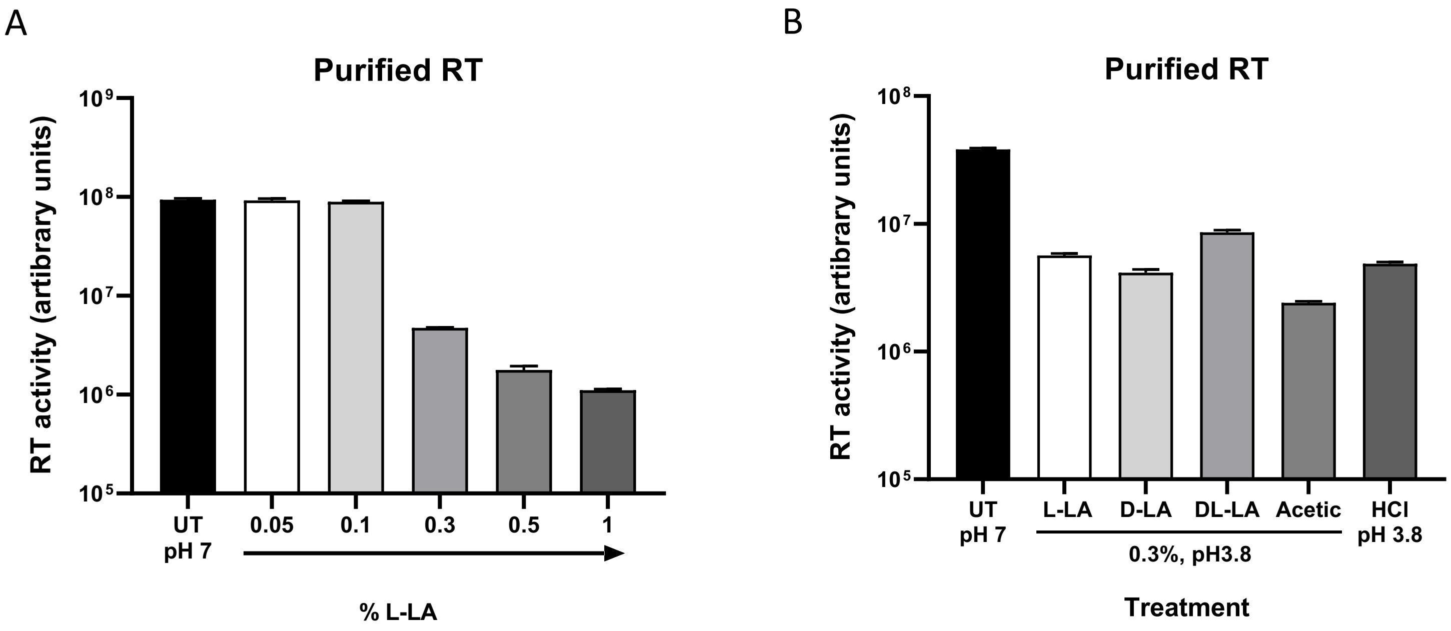

S4 Figure. Activity of purified reverse transcriptase (RT) after acid treatment. Purified recombinant HIV-1 RT was incubated with the indicated treatments for 2 min and RT activity determined after sample neutralization using ^{33}PdTTP substrate. (A) RT treated with increasing concentrations of L-lactic acid (L-LA) compared to untreated control (UT). (B) RT treated with L-lactic acid (L-LA), D-lactic acid (D-LA), racemic lactic acid (DL-LA), acetic acid and media acidified with HCl, all adjusted to pH 3.8. Error bars denote the mean ± SEM from n=6 technical replicates from one assay.
