# Supplementary material for: Lactic acid produced by optimal vaginal Lactobacillus spp. potently and specifically inactivates HIV-1 in vitro by targeting the viral RNA genome and reverse transcriptase
Source: PLoS Pathog. 2025 Oct 10;21(10):e1013594. doi: 10.1371/journal.ppat.1013594 (PMC12527216; doi:10.1371/journal.ppat.1013594)
Supplement: S1 Table — (PDF) [file ppat.1013594.s005.pdf]

S1 Table. Lactic acid, short chain fatty acids, and succinic acid concentrations representing an optimal vaginal microbiota and BV

Acid	Optimal vaginal microbiota (pH 3.8)	BV (pH 5.0)
DL-LA	100 mM	20 mM
Acetic	4 mM	100 mM
Propionic	1 mM	2 mM
Butyric	1 mM	2 mM
Succinic	1 mM	20 mM
HCl	Adjusted to pH 3.8	Adjusted to pH 5.0

BV, bacterial vaginosis; DL-LA, racemic mixture of D-lactic acid and L-lactic acid; HCl, hydrochloric acid
