# Supplementary material for: Lactic acid produced by optimal vaginal Lactobacillus spp. potently and specifically inactivates HIV-1 in vitro by targeting the viral RNA genome and reverse transcriptase
Source: PLoS Pathog. 2025 Oct 10;21(10):e1013594. doi: 10.1371/journal.ppat.1013594 (PMC12527216; doi:10.1371/journal.ppat.1013594)
Supplement: S2 Table — (PDF) [file ppat.1013594.s006.pdf]

S2 Table. SAXS data collection and scattering derived parameters

Data collection parameters	
Instrument	SAXS/WAXS Beamline at Australian Synchrotron
Beam geometry	120 micron point source
Wavelength (Angstrom)	1.033
q range (Angstrom – 1)	0.034 to 0.45
Exposure time (sec)	10x 1 second exposures with sample flow at 4 µl/sec
Concentration range (w/w)	0.01%, 0.05%, 0.1%, 0.3% and 1% of L-LA or D-LA
Temperature (K)	298

SAXS, Small Angle X-ray Scattering; WAXS, Wide Angle X-Ray Scattering; *q*, angle of the X-ray scatter; w/w, weight per weight; K, Kelvin
